# Supplementary material for: NF90 modulates processing of a subset of human pri-miRNAs
Source: Nucleic Acids Res. 2020 May 19;48(12):6874–88. doi: 10.1093/nar/gkaa386 (PMC7337520; doi:10.1093/nar/gkaa386)
Supplement: gkaa386_Supplemental_File [file gkaa386_supplemental_file.pdf]

A

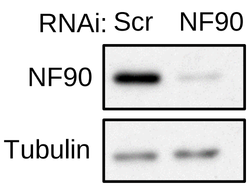

C

miRNAs	Upregulated	Downregulated
Mature	278	84
Precursors	217	77

B

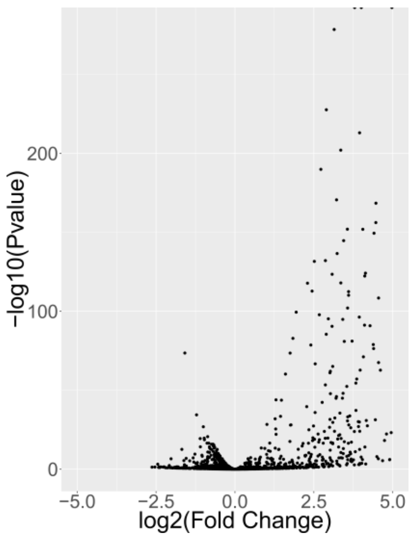

D

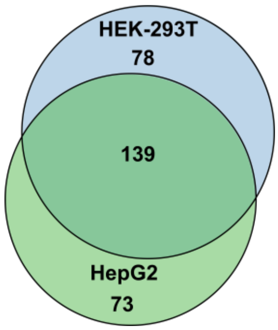

Figure S1. NF90 Modulates the Expression Level of miRNAs in HEK-293T cells. **(A)** Extracts of HEK-293T cells transfected with siRNA targeting NF90 or a non-targeting control (Scr) were analyzed by Western blot using the indicated antibodies. **(B)** Samples described in A were analyzed by small RNA-seq. Results are shown as log2 fold change versus –log10 p-value. **(C)** Table summarizing the number of mature miRNAs and pri-miRNAs modulated in HEK-293T cells upon loss of NF90, according to small-RNA seq. **(D)** Venn diagram representing the number of miRNAs upregulated following knock-down of NF90 in HepG2 versus HEK-293T cells.

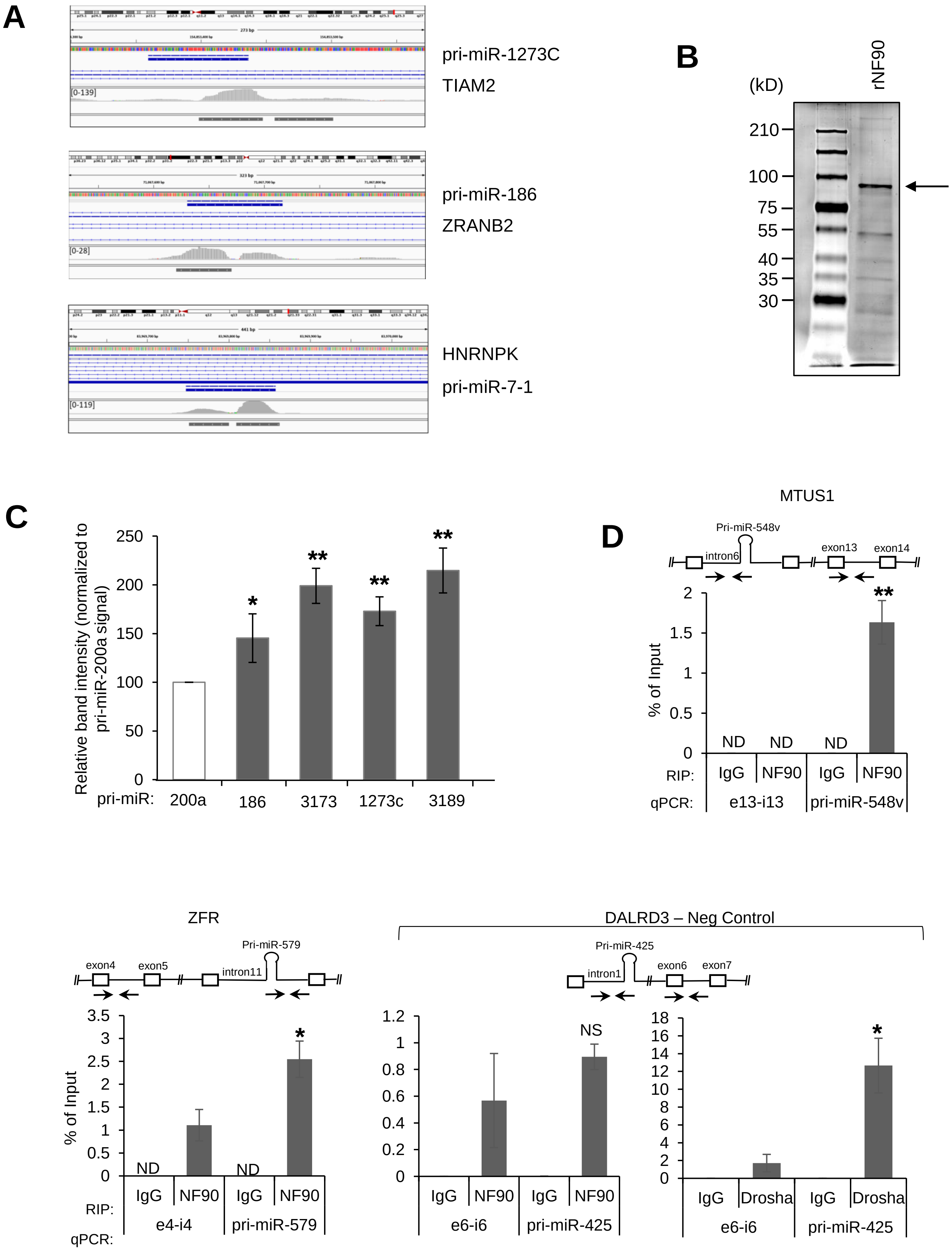
A
pri-miR-1273C
TIAM2
[0-139]
pri-miR-186
ZRANB2
[0-28]
HNRNPK
pri-miR-7-1
[0-119]
B
rNF90
(kD)
210
100
75
55
40
35
30
C
Relative band intensity (normalized to pri-miR-200a signal)
250
200
150
100
50
0
*
**
**
**
pri-miR:
200a
186
3173
1273c
3189
MTUS1
D
Pri-miR-548v
intron6
exon13
exon14
% of Input
2
1.5
1
0.5
0
ND
ND
ND
**
RIP:
IgG
NF90
IgG
NF90
qPCR:
e13-i13
pri-miR-548v
ZFR
Pri-miR-579
exon4
exon5
intron11
3.5
3
2.5
2
1.5
1
0.5
0
% of Input
ND
ND
*
IgG
NF90
IgG
NF90
RIP:
e4-i4
pri-miR-579
qPCR:
DALRD3 – Neg Control
Pri-miR-425
intron1
exon6
exon7
1.2
1
0.8
0.6
0.4
0.2
0
NS
IgG
NF90
IgG
NF90
e6-i6
pri-miR-425
18
16
14
12
10
8
6
4
2
0
*
IgG
Drosha
IgG
Drosha
e6-i6
pri-miR-425

Figure S2. (A) Browser shots of NF90 eCLIP read coverage over the pri-miRNAs indicated on the figure. Blue lines represent host gene showing localization of the pri-miRNA. eCLIP reads are shown in grey and locations of eCLIP peaks are shown as dark grey bars. The arrows indicate the strand from which the reads originated. **(B)** Recombinant NF90 used in RNA EMSA was analyzed by SDS-PAGE and Comassie brilliant blue staining. **(C)** RNA EMSA shown in Figure 2C performed using recombinant NF90 and probed with radiolabeled pri-miRNAs was carried out in three independent experiments. The graph shows the mean ± SD of the relative band intensity normalized to pri-miR-200a signal (*$P < 0.05$ **$P < 0.005$, *independent Student's t test*).

(D) HepG2 cells transfected with siRNA targeting NF90 or a non-targeting control (Scr) were subjected to RIP using anti-NF90, anti-Drosha or a control antibody. Immunoprecipitates were analyzed by RT-qPCR. ND indicates 'Not Detected'. Data represent mean ± SEM obtained from 3 independent experiments (*$P < 0.05$, **$P < 0.01$, *independent Student's t test*).

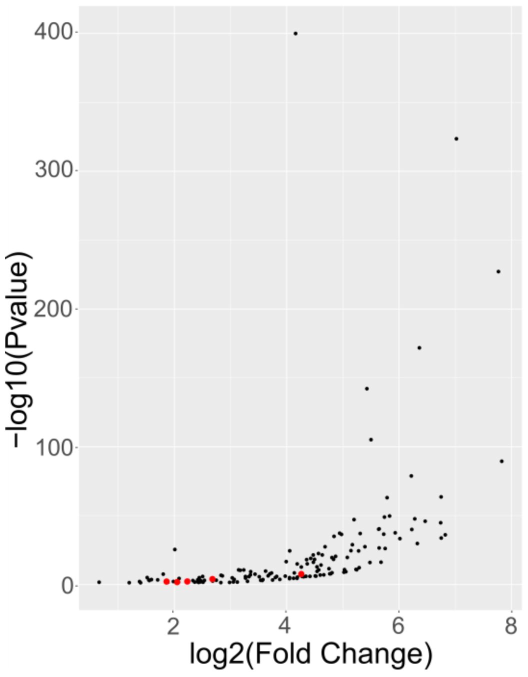

Figure S3. DotPlot of Drosha-associated pri-miRNAs, determined by eCLIP analysis. Red dots indicate the position of pri-miRNAs that are also positive for association with NF90.

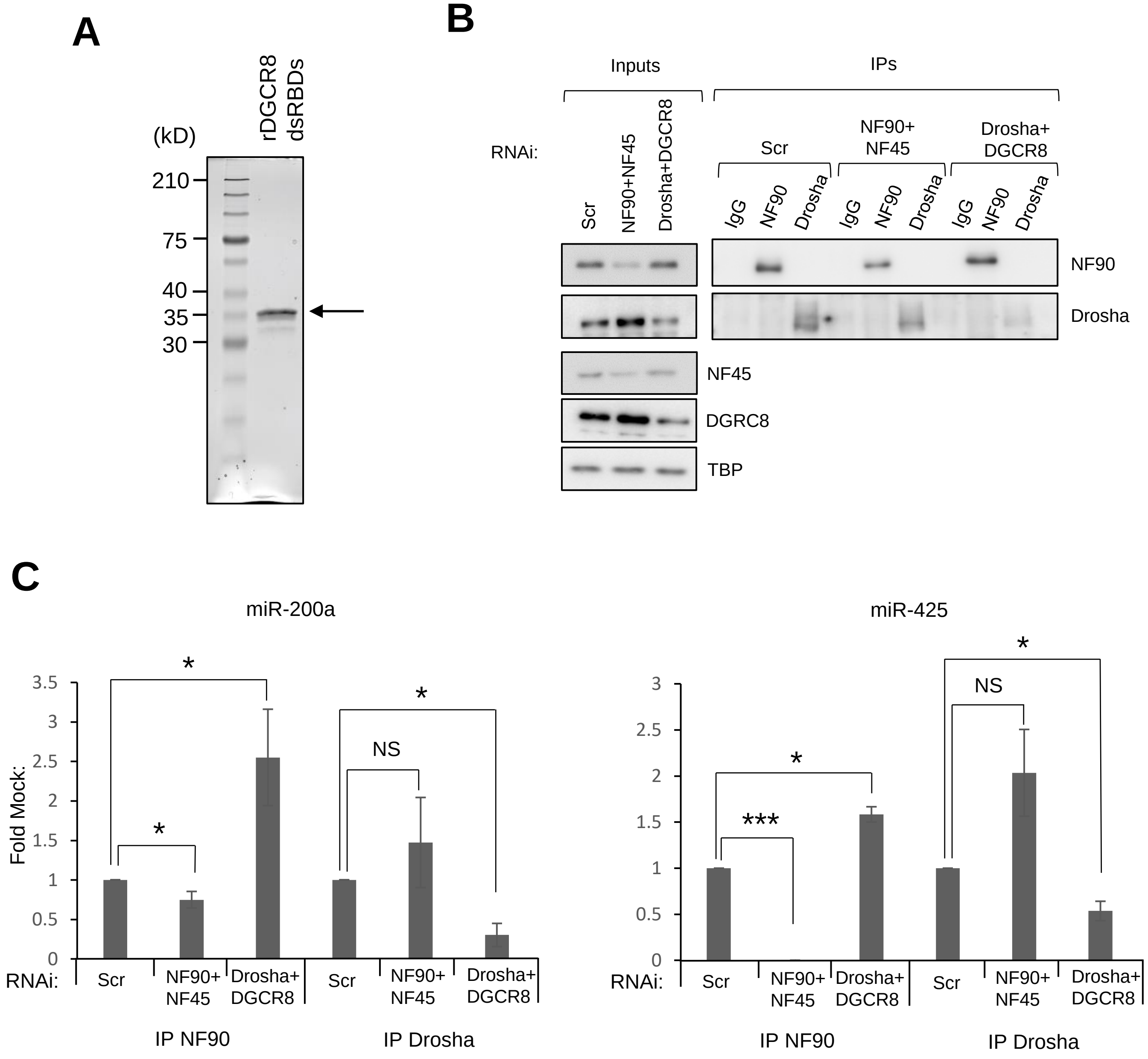

Figure S4. **(A)** Recombinant DGCR8 dsRBDs used in RNA EMSA was analyzed by SDS-PAGE and Comassie brilliant blue staining. **(B)** Extracts of HepG2 cells transfected with siRNAs targeting NF90 and NF45, Drosha and DGCR8 or a non-targeting control (Scr) were analyzed by Western blot using the indicated antibodies (left panel). The same extracts were used of RIP using antibodies anti-NF90, anti-Drosha or control IgG, as indicated (right panel). **(C)** HepG2 cells transfected with siRNA targeting NF90 and NF45 or Drosha and DGCR8 or a non-targeting control (Scr), as indicated, were subjected to RIP using anti-NF90, anti-Drosha or a control IgG antibody. Immunoprecipitates were analyzed by RT-qPCR. NS indicates 'Not Significant'. Data represent Fold mock (IgG) relative to the control sample (siScr), which was attributed a value of 1, obtained from 3 independent experiments *(*P < 0.05, ***P < 0.001, independent Student's t test)*.

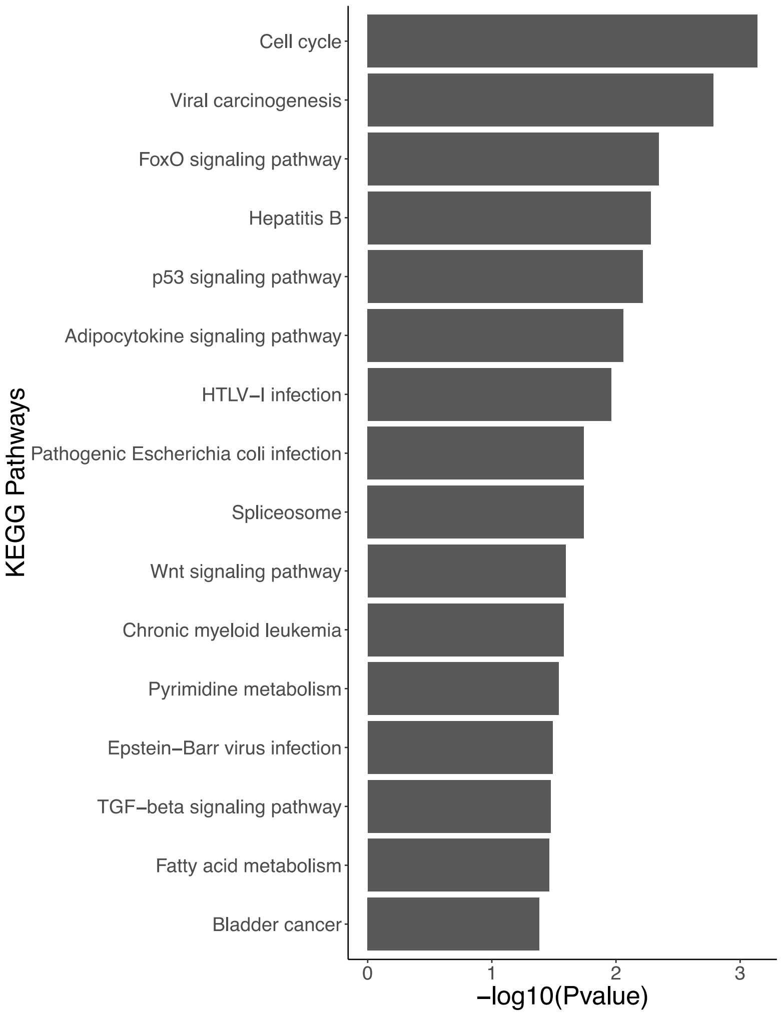

Figure S5. NF90 double positive pri-miRNAs target genes involved in viral infection and cancer. Gene ontology of validated targets of NF90-bound and upregulated miRNAs.

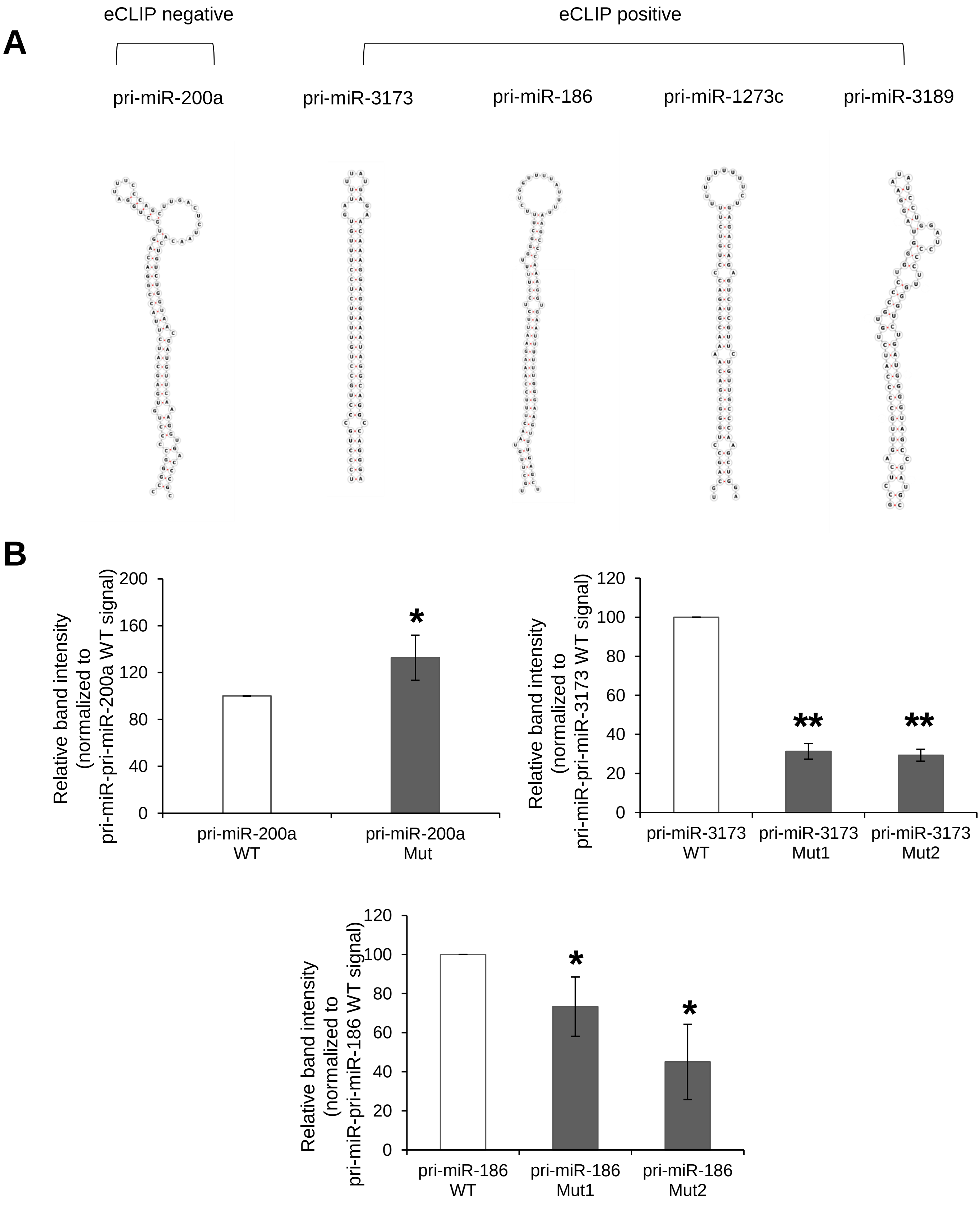

Figure S6. (A) NF90-associated pri-miRNAs are highly stable. Predicted folding of pri-miRNAs that are significantly associated or not with NF90 as indicated. RNA structures were predicted using FORNA. **(B)** RNA EMSA shown in Figure 5B performed using recombinant NF90 and probed with radiolabeled WT or mutant pri-miRNAs was carried out in three independent experiments. The graph shows the mean ± SD of the relative band intensity normalized to the WT signal *(*P < 0.05 **P < 0.005, independent Student's t test).*

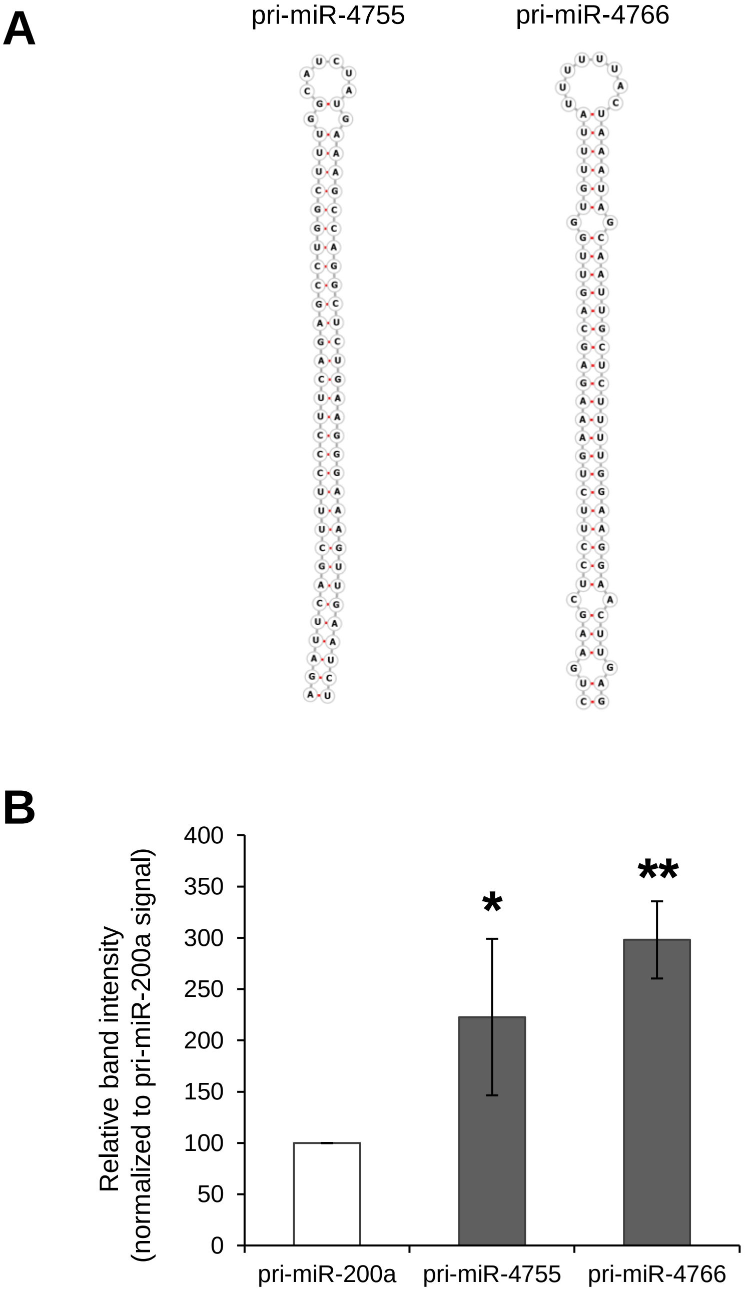

Figure S7. (A) NF90-modulated pri-miRNAs are highly stable. Predicted folding of examples of pri-miRNAs whose mature products are upregulated following loss of NF90. RNA structures were predicted using FORNA. **(B)** RNA EMSA shown in Figure 6C performed using recombinant NF90 and probed with radiolabeled pri-miR-200a, pri-miR-4755 and pri-miR-4766 was carried out in three independent experiments. The graph shows the mean ± SD of the relative band intensity normalized to pri-miR-200a *(*P < 0.05, **P < 0.05, independent Student's t test).*

Supplementary Table S1. Double stranded siRNAs used in this study.

siRNA	Sequence (5' to 3')
Scr	gcgcgcuuuguaggauucg(dTdT)
Scr#2	ucugcaagguuaggcgucu(dTdT)
NF90	ccaaggaacucuaucacaa(dTdT)
NF90#2	gaguugaaguauugauaac(dTdT)
Drosha	cgaguaggcuucgugacuu(dTdT)
DGCR8	caucggacaagagugugau(dTdT)
NF45	guggugauacucaagauucugccaa(dTdT)

Supplementary Table S2. Primary antibodies used in this study.

Antibody	Reference	Supplier
NF90	A303-651A	Bethyl Laboratories
GDF-15	sc-377195	SCBT
ZRANB2	sc-514200	SCBT
TIAM2	sc-514090	SCBT
NDUFS8	sc-515527	SCBT
TUBULIN	DM1A clone, T6199	Sigma-Aldrich
TBP	sc-421	SCBT
Drosha (IP)	Ab-12286	Abcam
Drosha (WB)	sc-33778	SCBT
DGCR8	Ab-82876	Abcam
Rabbit IgG	p120-101	Bethyl Laboratories
DICER1	sc-136981	SCBT
ZFR	A14281	ABClonal
NF45	A303-147A	Bethyl Laboratories

Supplementary Table S3. Primers used in this study.

Primer	Forward (5' to 3')	Reverse (5' to 3')
Spiced GAPDH	cac atc gct cag aca cca t	gag gtc aat gaa ggg gtc at
U6	ctc gct tcg gca gca cat ata c	gga acg ctt cac gaa ttt gcg tg
pri-miR-1273C (EMSA)	ctt ggg aag ctg agg tag gc	act tgg tac tga ggc gga gg
pri-miR-186 (EMSA)	aca gaa cac cca tca tat tc	gtt gac att cac atg ctt c
pri-miR-200a (EMSA)	ctg gct gct cac cgc tcc	gat gtg cct cgg tgg tgt cc
pri-miR-3173 (EMSA)	cat tgg agg tct agg gct ta	gtt ctt cct cgg cac aag
pri-miR-3189 (EMSA)	agc agc ccc cat atc taa tc	ctg gca tcc ctg tac ctc
pri-miR-4755 (EMSA)	aga gat gag gaa ggt tat ggc t	tgg ccc aaa cct cat aga c
pri-miR-4766 (EMSA)	ccc ttc tac ctt tct gaa gct c	cac aca ggt ggc act caa c
5'RLM-RACE pri-miR-186 (outer)	gct gat ggc gat gaa tga aca ctg (adapter)	aaa cca ggt ata tgg cac agc aac
5'RLM-RACE pri-miR-186 (inner)	cgc gga tcc gaa cac tgc gtt tgc tgg ctt tga tg (adapter)	tgt tga cat tca cat gct tca ggt
5'RLM-RACE pri-miR-3189 (outer)	gct gat ggc gat gaa tga aca ctg (adapter)	acc aca ccc cca ttg ttt ctct
5'RLM-RACE pri-miR-3189 (inner)	cgc gga tcc gaa cac tgc gtt tgc tgg ctt tga tg (adapter)	acc aca ccc cca ttg ttt ctct
ZRANB2 e3-i3 (RIP)	gag ccg agg cct att tag tg	aag gtt acc ctg gct tgt ca
ZRANB2 pri-miR-186 (RIP)	cct gaa gca tgt gaa tgt caa	cca ggt ata tgg cac agc aa
DICER i22-e23 (RIP)	ggc cat gat ttt aaa gtt gc	tcc tcc tcc tcg taa tcc tc
DICER pri-miR-3173 (RIP)	aac aga acc tgg aca ctg ag	aga cac caa cct cac tca ag
TIAM2 i12-e13 (RIP)	ggt ttg agt ttg cag cct tc	aga aaa cag ggc ctc cat ct
TIAM2 pri-miR-1273c (RIP)	ctg aaa tgc tgt ccc cat ct	tgc cca gtc tct tct cgt tt
GDF-15 i2-e3 (RIP)	ctc cca aag tgc tgg gat ta	aga gat acg cag gtg cag gt
GDF-15 pri-miR-3189 (RIP)	acg cta cga gga cct gct aa	tta gat atg ggg gct gct tg
MTUS1 e13-i13 (RIP)	aga aag cct gaa agc tgt gtt	aat gca ggg ctc aat ttc ac
MTUS1 pri-miR-548v (RIP)	tct cag cgt ggc tac tag gaa	tgt acg gct aca gca tct gg
ZFR e4-i4 (RIP)	tca gcc ttc tgt tgc tga aa	ggg ctg aaa gtc cag aaa tg
ZFR pri-miR579 (RIP)	ttt tgt gtc tgg cat cgt tc	gga aac aag ttg cat gtc ca
DALRD3 e6-i6 (RIP)	atc tgt ggc cct gtg aaa gt	gta tgc cgg aac ctg tgt tt
DALRD3 pri-miR-425 (RIP)	agg gct gca atg gta gtg ac	aag gtg cat gac ctg gag ac
NDUFS8 miRNA unspliced	ctt agc cgg agt cca gga g	aag ccg cag tag atg cac tt
NDUFS8 miRNA spliced	cca cca tca act acc cgt tc	aag ccg cag tag atg cac tt
NDUFS8 unspliced	caa tgg cag cgt cct aca gt	caa cgg gga cac cac act
NDUFS8 spliced	atg gca gcg tcc tac agt g	agc agc ata ggc gtg gtg
TIAM2 miRNA unspliced	aac caa ggt ttt gcg tga ag	acc cag gta gct gaa gac ga
TIAM2 miRNA spliced	gct cag cca cca cct ata cc	acc cag gta gct gaa gac ga
TIAM2 unspliced	gga tgc ttt gga tag ccg ta	cga atg tgt gga ttc act tc
TIAM2 spliced	atc agt gac tgg acg gga ag	tcg cat gtg tgg att cac tt
ZFR miRNA unspliced	gat gca agt tct tgg gct gt	cct gga gga cca tga gga ta
ZFR miRNA spliced	gga gta ctg gcg aag acg ag	cct gga gga cca tga gga ta
ZFR unspliced	tcc ttc cac ttg tct cat agc a	ttt cag caa cag aag gct ga
ZFR spliced	tta tgg agg cta ccc cac tg	cag cag ttg ctg ttg gtt gt
DICER1 miRNA unspliced	gga aga gtt tga atg gct ca	ggg ctt ttc att cat cca gtg
DICER1 miRNA spliced	gtc cga tgg ttc tcg aag	gtt cta gca cag ctt act g
DICER1 unspliced	aat tgc att ctc act act gca	gtc cac aat cca cca caa tc
DICER1 spliced	att gtc cat cat gtc ctc gc	gtc cac aat cca cca caa tc

Supplementary Table S4. Wild-type and mutant pri-miRNAs sequences used for RNA EMSA. The pri-miRNA sequence is shown in red and flanking sequence is shown in black.

	pri-miR-200a	pri-miR-3173	pri-miR-186
Wild-type	ctggctgctcaccgctccggttcttccctgggct<br>tccacagcagcccctgcctgcctggcgggac<br>cccacgtccctcccgggcccctgtgagcatct<br>taccggacagtgctggatttcccagcttgactc<br>taacactgtctggtaacgatgttcaaaggtga<br>cccgccgctcgccggggacaccaccgagg<br>cacatc	cattggaggtctagggcttattttccagat<br>agaattgagtctttgttggtcttgggccag<br>cttccctgccctgcctgttttctcctttgtgatt<br>ttatgagaacaaaggaggaaataggca<br>ggccagggaaacgatctctctccctctctt<br>gtgccgaggaagaact	acagaacacccatcatattcttcccaaacattttttcat<br>tgcttgtaactttccaaagaattctccttttgggctttctg<br>gttttattttaagcccaaaggtgaatttttgggaagttt<br>gagctaaattccttcaaccaaaatatacaagtgaag<br>aaaaaaaatttgtatttaaacatttgcacatttacttct<br>acctgaagcatgtgaatgtcaac
Mutant #1	ctggctgctcaccgctccggttcttccctgggct<br>tccacagcagcccctgcctgcctggcgggac<br>cccacgtccctcccgggcccctgtgagcatct<br>taccggacagtgctggatttcccagcctgtctg<br>gtaacgatgttcaaaggtgacccgccgctcg<br>ccggggacaccaccgaggcacatc	cattggaggtctagggcttattttccagat<br>agaattgagtctttgttggtcttgggccag<br>cttccctgcgacgcctgcctgttttctccttt<br>gtgattttatgagaacaaaggaggaaag<br>cgctaggcaggccagggaaacgatctc<br>tctccctctcttgtgccgaggaagaact	acagaacacccatcatattcttcccaaacattttttcat<br>tgcttgtaactttccaactaaagaattctccttttgggct<br>ttctggttttattttaagcccacctctaaggtgaatttttg<br>ggaagtttgagctaaattccttcaaccaaaatataca<br>agtgaagaaaaaaaatttgtatttaaacatttgcaca<br>tttacttctacctgaagcatgtgaatgtcaac
Mutant #2		cattggaggtctagggcttattttccagat<br>agaattgagtctttgttggtcttgggccag<br>cttccctgcaagtcctgttttctcctttgtgatt<br>ttatgagaacaaaggagaccctaggca<br>ggccagggaaacgatctctctccctctctt<br>gtgccgaggaagaact	acagaacacccatcatattcttcccaaacattttttcat<br>tgcttgtccagttccaaagaattctccttttgggctttct<br>ggttttattttaagcccaaaggtgaaccacgtgggaa<br>gtttgagctaaattccttcaaccaaaatatacaagtg<br>aagaaaaaaaatttgtatttaaacatttgcacatttac<br>ttctacctgaagcatgtgaatgtcaac

Supplementary Table S5. NF90-associated pri-miRNAs, as determined by eCLIP analysis.

hsa-mir-1273c	hsa-mir-4485	hsa-mir-548d-1
hsa-mir-1290	hsa-mir-4635	hsa-mir-548u
hsa-mir-15b	hsa-mir-4659a	hsa-mir-548v
hsa-mir-186	hsa-mir-4687	hsa-mir-5581
hsa-mir-1914	hsa-mir-4712	hsa-mir-570
hsa-mir-3140	hsa-mir-4714	hsa-mir-578
hsa-mir-3145	hsa-mir-4730	hsa-mir-579
hsa-mir-3173	hsa-mir-4762	hsa-mir-606
hsa-mir-3189	hsa-mir-4775	hsa-mir-624
hsa-mir-3646	hsa-mir-4779	hsa-mir-6751
hsa-mir-3648-1	hsa-mir-4782	hsa-mir-6839
hsa-mir-3680-1	hsa-mir-548aq	hsa-mir-7-1
hsa-mir-3939	hsa-mir-548ar	

Supplementary Table S6. 'Double positive' miRNAs whose abundance increased following loss of NF90 and that were positive for NF90 association by eCLIP, and their host gene.

miRNA	Small RNA-seq		Host Gene
	Fold Change (log2)	p value (-Log10)	
hsa-mir-1273c	3.55	33.94	TIAM2
hsa-mir-186	1.01	4.55	ZRANB2
hsa-mir-3140	3.77	37.16	FBXW7
hsa-mir-3145	2.56	9.78	NHSL1
hsa-mir-3173	3.03	25.36	DICER1
hsa-mir-3189	2.16	19.36	GDF15
hsa-mir-3646	3.76	4.88	HNF4A
hsa-mir-3939	1.27	7.59	RP1-167A14.2
hsa-mir-4659a	2.21	10.93	AGPAT5
hsa-mir-4714	3.36	23.31	IGF1R
hsa-mir-4762	2.14	5.66	ATXN10
hsa-mir-4775	1.37	3.72	CCNYL1
hsa-mir-4779	4.03	10.9	IMMT
hsa-mir-4782	3.94	3.87	SLC35F5
hsa-mir-548ar	3.24	6.79	CDC16
hsa-mir-548u	2.24	2.38	PRIM2
hsa-mir-548v	2.01	7.81	MTUS1
hsa-mir-5581	2.69	19.23	MEAF6
hsa-mir-578	2.17	3.18	CPE
hsa-mir-579	4	73.29	ZFR
hsa-mir-624	2.68	25.49	STRN3
hsa-mir-7-1	0.99	5.76	HNRNPK

Supplementary Table S7. MiRNAs downregulated in abundance following loss of NF90 and that are associated with NF90 by eCLIP.

miRNA	Fold Change (log2)	p value (-Log10)	Host Gene
hsa-mir-1914	-0.86	2.88	UCKL1
hsa-mir-6751	-1.52	3.73	SYVN1
